# Development of Multilayer Transducer and Omnidirectional Reflection Model for Active Reflection Control

**DOI:** 10.3390/s23010521

**Published:** 2023-01-03

**Authors:** Beom Hoon Park, Han Bin Choi, Hee-Seon Seo, Yub Je, Hak Yi, Kwan Kyu Park

**Affiliations:** 1 Department of Convergence Mechanical Engineering, Hanyang University, Seoul 04763, Republic of Korea; 2 Maritime Technology Research Institute, Agency for Defense Development, Changwon 51682, Republic of Korea; 3 Department of Mechanical Engineering, Kyungpook National University, Daegu 41566, Republic of Korea

**Keywords:** cymbal transducer, stacked piezoelectric transducer, active reflection control, SONAR, piezo material, unmanned underwater vehicle (UUV)

## Abstract

Underwater detection is accomplished using an underwater ultrasonic sensor, sound navigation and ranging (SONAR). Stealth to avoid detection by SONAR plays a major role in modern underwater warfare. In this study, we propose a smart skin that avoids detection by SONAR via controlling the signal reflected from an unmanned underwater vehicle (UUV). The smart skin is a multilayer transducer composed of an acoustic window, a double-layer receiver, and a single-layer transmitter. It separates the incident signal from the reflected signal from outside through the time-delay separation method and cancels the reflected wave from the phase-shifted transmission sound. The characteristics of the receiving and transmitting sensors were analyzed using a finite element analysis. Three types of devices were compared in the design of the sensors. Polyvinylidene fluoride (PVDF), which had little effect on the transmitted sound, was selected as the receiving sensor. A stacked piezoelectric transducer with high sensitivity compared to a cymbal transducer was used as the transmitter. The active reflection control system was modeled and verified using 2D 360° reflection experiments. The stealth effect that could be achieved by applying a smart skin to a UUV was presented through an active reflection–control omnidirectional reflection model.

## 1. Introduction

Sound navigation and ranging (SONAR) is a sensor that detects signals radiated or reflected from objects in the water. Most underwater detection is performed by SONAR, which is used for various purposes, such as fish location detection, seabed detection, and military purposes. During World War I, the importance of SONAR for detecting U-boats was highlighted. As the detection of submarines became important during World War II, research on SONAR was actively conducted. As such, in modern underwater combat, SONAR for underwater detection is essential, and the research to improve its performance is progressing. In response to the growing significance of SONAR in underwater detection technology, the stealth technique, which avoids SONAR detection, has also emerged as a major topic. Research on reflection control is in progress to reduce the SONAR signal.

The pulse-echo-type active SONAR identifies an object based on the signal level and receiving time. Stealth techniques have been studied to control pulse-echo signals. Passive sound absorbing methods and active reflection control methods exist for controlling the reflection signals. The passive acoustic structure is the most commonly used method for sound absorption. A passive acoustic structure uses the high-damping properties of sound absorbers or converts acoustic energy into thermal energy. This method is inexpensive and convenient. Cheng [1] studied the sound absorption effect of a porous aluminum structure. The optimized porosity for aluminum structures with pores having 2–5 mm diameter was presented. It has also been proven that the sound absorption effect is better in thick samples than in thin ones. Non-metallic porous materials, such as polyurethane or polyolefins, have also been studied for sound absorption in water [2]. As for metallic materials, the size of the porous material and the thickness of the sound-absorbing layer influence the sound-absorbing effect. Polyurethane has better sound-absorption performance in the mid-frequency bandwidth than polyolefins [3]. Porous materials have a major drawback of impedance mismatch despite good sound absorption; hence, there are a few studies conducted that directly utilize porous materials. A sound-absorbing structure is also formed by the scattering of sound waves in a certain pattern [4]. It had a wedge-shaped structure with butyl rubber. Super-ellipsoidal and spiral neck structures have also been studied for absorption at low frequencies [5]. The metamaterial is designed using a tungsten disk. The higher the density of the disc, the better the sound absorption is [6]. Several studies have been conducted, and their performance has been proven. However, the passive sound absorption method for sound absorption in the low-frequency band still has the disadvantage of being thick or difficult to use in underwater vehicles.

A study to increase sound absorption efficiency by changing the control technology and structure has been presented [7]. In the duct of 1.2 m, the low-frequency sound was also canceled through the feedback process. It is a method in which the signal received by the microphone 77.5 cm away from the noise speaker is offset by the control speaker located 31.5 cm away [8]. A study on passive–active absorption using a micro-perforated panel and a loudspeaker was also conducted [9]. The effect of increasing the passive absorption rate from 0.2 to 0.72 was demonstrated. In addition, passive–active sound absorption for oblique incident sounds was also studied. The same perforated panel was used, and sound absorption was possible up to a maximum angle of incidence of 20 degrees [10]. A curved PVDF has been studied for passive–active noise radiation control [11]. Smart foam has also been developed for passive–active noise radiation control. The smart foam composed of polyurethane for passive control and PVDF for active control controlled the radiated sound of the piston and attenuated 10 dB on average. There is a study that utilized 10 microphones in the form of a hemisphere and controlled sound radiation with adaptive feedforward and feedback methods using smart foam. The active cell was composed of curved PVDF and attenuated up to 17.6 dB [12]. A smart structure for active control of the cylinder structure has also been proposed. The smart cylinder was modeled with Hamilton’s principle and Sanders shell theory and assumptions. A smart structure was fabricated using a transceiver sensor composed of PZT. The simulation and experiment were similar, and the radiated pressure was reduced by up to 10 dB [13]. Algorithms and transducers for active control have been studied in various ways. It showed a more advanced performance but was performed in air rather than underwater. Because the underwater environment is different, the wavelength changes and the size of the transducer is also different.

Active control of sound reflection using multilayer transducers has also been analytically modeled. It consists of a total of 10 layers, and there are sensors and actuators made of piezocomposite layers [14]. The transducer of the above structure was fabricated, and the performance test was conducted using a pulse tube. When it was uncontrolled, it showed a decrease of 2–3 dB, and when it was controlled, the sound absorption performance was around 20 dB [15]. Among the studies of analytic active sound absorbers, 1–3 composites were used. Due to the thickness limitation, the passive sound absorption effect was low at low frequencies. The sound absorption effect is enhanced by adjusting the resistance and capacitance of the external circuit [16]. A smart structure with a thickness of less than 50 mm was designed using active control by a single unit [17]. In addition to using the characteristics or structure of the material, the system was configured for sound absorption. The system consists of two receiving sensors and one actuator. When a sound wave is incident from outside, the receiving layer detects the signal. The detected signal is subjected to active reflection control through processes such as reflection calculation, actuator sensitivity, and attenuation, using an antiphase [18]. To construct the system, tonpilz transducers were used as actuators, and two receiving layers were designed using polyvinylidene fluoride PVDF [19]. The system exhibited an attenuation performance of 30 dB at low frequencies of 4–11 kHz for normal incident signals. This was performed for the normal-incidence signals inside the tube.

Various methods and sensors have been studied for reflection control. However, passive control is thick and difficult to absorb at low frequencies. In order to supplement the limitations of the passive method, passive-active reflection control has been proposed. High and medium frequencies are absorbed by passive control, and low frequencies are controlled by active reflection with a wide bandwidth transducer. Most of the proposed methods consist of a single channel or have large transducers. Additionally, the thickness is more than 10 cm, which is not thin enough for UUV application. To apply the smart structure to UUV, the thickness must be thin, and at the same time, the small-sized structure must be applied in the form of an array to absorb sound even from an oblique incident signal. In addition, the transducers must be configured as an array to enable active reflection control not only for normal incident signals but also for oblique incident signals. Therefore, the diameter of the transducer should be smaller than half the wavelength and the thickness should be thin to minimize the effect on the movement of UUV.

In this study, we developed a smart skin for active reflection control and analyzed its effects using an omnidirectional reflection model. The smart skin is designed as a multilayer, including two receiving sensors, one transmitting sensor, and an acoustic window for active reflection cancellation. The influence of the receiver on the transmitted sound was calculated using finite element analysis. The transmitter was designed using a cymbal transducer and a stacked piezoelectric ultrasound transducer, and their characteristics were compared. An active reflection control analysis model was developed and verified through a 2D reflection pattern experiment. Finally, the effect of the smart skin was presented through a 3D omnidirectional reflection analysis model.

## 2. Materials and Methods

### 2.1. Multi-Layer Transducer Design and Modeling

#### 2.1.1. Double-Layer Receiver to Distinguish between Incident and Reflected Signal

**Figure 1:**
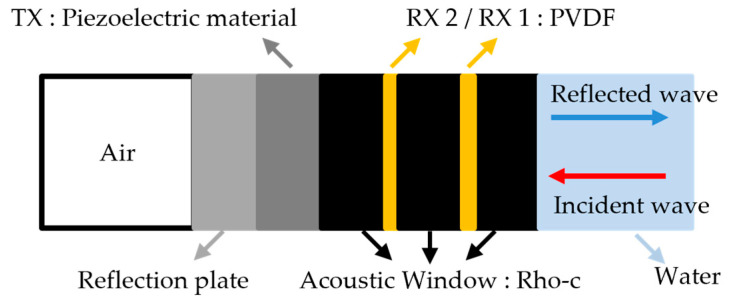
Schematic of smart skin.

**Figure 2:**
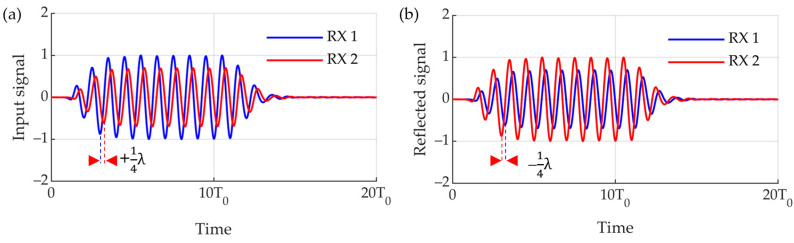
Time delay separation method. (**a**) Time delay of RX1 and RX2 in case of incident wave (**b**) time delay of RX1 and RX2 in case of reflected wave.

**Figure 3:**
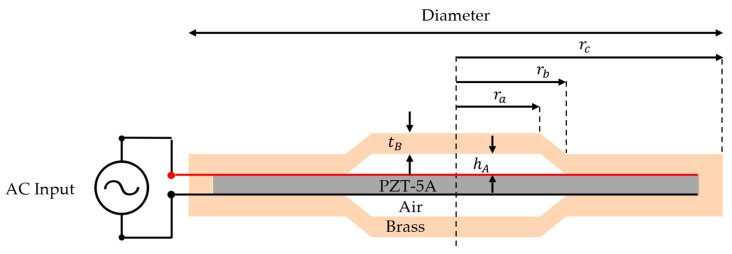
Schematic of cymbal transducer.

Active SONAR detects targets by emitting sound waves and receiving the reflected signals back. Active SONAR transmits and receives sound waves from a single sensor or separates the transmitting and receiving sensors. Both methods emit and reflect acoustic waves to reconstruct the acoustic signal measured by the receiver in order to detect the position of the object. In other words, if the reflected signal can be controlled, it may not be detected by the SONAR. Active reflection control is a technology that receives and analyzes acoustic waves emitted from SONAR and cancels the reflected waves by transmitting a signal whose phase is opposite. Therefore, it must consist of a receiver and a transmitter [20]. In particular, the receiver must distinguish between an externally incident signal and the reflected and outgoing reflected waves. For this purpose, a double-layer receiver was designed (Figure 1). The two-layer receiving sensor used the delay-separation technique to distinguish the incident sound from the reflected sound (Figure 2). The active reflection-control signal of the transmitter passes through the receiver (Figure 3). If the acoustic impedance of the receiver is large, the transmission is reflected owing to impedance mismatch. Transmission resonates inside the multilayer transducer, causing a ring down and degrading the quality. Therefore, the acoustic impedance of the receiver should be lowered to minimize its effect on the transmitter propagation. The effects of lead zirconate titanate (PZT) with thicknesses of 1 mm and 0.5 mm and those of PVDF with a thickness of 110 µm were compared by finite element analysis.

#### 2.1.2. Design and Modeling of Cymbal Transducer

If the pitch of the transducer array is greater than half the wavelength, the lobes of the array elements interfere, and a grating lobe is formed. Because the grating lobes can cause side effects, the pitch between the elements and the width of the elements are limited to reduce the grating lobes. Therefore, to lower the frequency within a limited size, a cymbal transducer and stacked piezoelectric transducer were designed, and their characteristics were compared.

**Figure 4:**
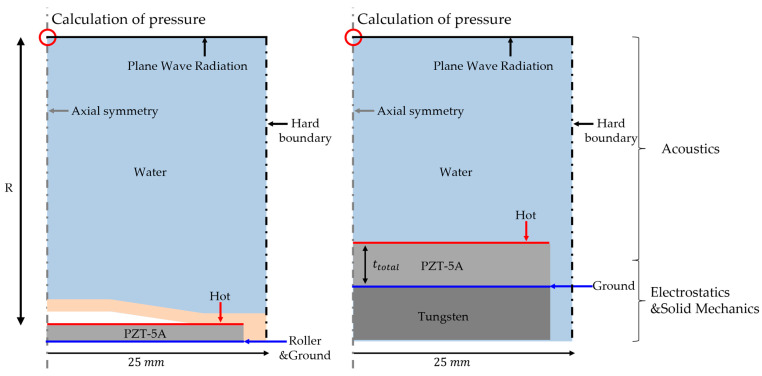
Finite element method (FEM) model of cymbal transducer and stacked PZT transducer.

The cymbal transducer is a miniature transducer with a simple structure composed of a piezo ceramic and metal cap (Figure 3) [21]. Its simple structure renders it easy to be manufactured, with the advantage of reduced cost and time required for manufacturing. These characteristics make them good candidates for elements of ultrasonic transducer arrays. The cymbal transducer was modeled using COMSOL Multiphysics Version 5.2, a multiphysics finite element analysis program, to calculate the effects of each parameter on the center frequency, bandwidth, and sensitivity (Figure 4).

Three physical models were applied to the finite element analysis model. Electrostatics induce a piezoelectric effect by forming an electric field generated by the electrical input in the PZT. In solid mechanics, the thickness mode vibration of PZT is analyzed by the electrostatic force generated by the piezoelectric effect. The movement of the PZT acted as a displacement on the metal cap and vibrated in the bending mode. Finally, the acoustic method was used to calculate the sound pressure caused by the vibration of the metal cap. Cymbal transducers typically use cylindrical PZT disks and metal caps. Therefore, they were modeled with an axial symmetry condition, and boundary conditions, such as a hard boundary, to simulate the array and plane wave radiation for far-field applications were utilized. Because the cymbal transducer has a symmetrical structure in the upper and lower layers, the roller condition was applied to reduce the amount of analysis. The PZT material used was PZT-5A (PZT-5A, Piezo.com, Division of Mide Technology, Woburn, MA, USA), and the metal cap was brass. Because it is an underwater ultrasonic transducer, the medium is water, and the length R of the medium is 1 m for calculating the transmitting voltage response (TVR). Five parameters were used in the design of the cymbal transducer: $t_{b}$: thickness of the metal cap, $h_{A}$: height of the air layer, $r_{a}$: radius of the metal cap, $r_{b}$: radius of the cavity base, and $r_{c}$: total radius represent the radii of the transducer, limited to half the pitch (=1/4 of the wavelength). Therefore, parameter sweeps were performed for tm, $h_{a}$, $r_{a}$, and $r_{b}$.

#### 2.1.3. Design of Stacked Piezo-Electric Transducer

**Figure 5:**
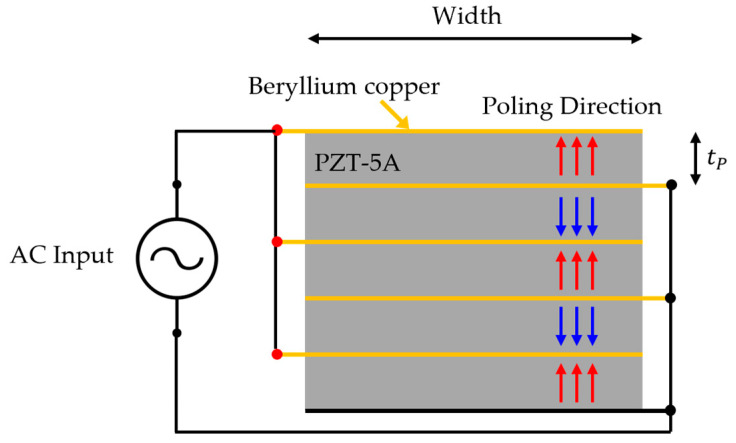
Schematic of stacked piezoelectric transducer.

Piezoelectric materials generally vibrate in the thickness mode, and the frequency is mainly determined by the thickness. The lower the frequency, the longer the wavelength; thus, the thickness of the piezoelectric material increases. However, it is challenging to fabricate PZT at the centimeter level. To compensate for this, stacked piezoelectric transducers have been proposed [22]. In the PZT-5A stack process, a low-viscosity epoxy (Epo^®^-Tek 301, Epoxy Technology, Billerica, MA, USA) was used to minimize its influence on the vibration mode of PZT. An electrode was placed between the PZT and epoxy to maintain the electric field force while stacking the piezoelectric material. Changing the poling direction of the PZT according to the order of the hot ground is advantageous for improving sensitivity (Figure 5). $t_{p}$ is the thickness of the single PZT.

In the analytical model of the stacked piezoelectric transducer, the acoustic boundary conditions were the same as those in the cylindrical transducer model. There is a difference between electrostatic and solid mechanics. In a stacked piezoelectric transducer, only tens of centimeters thick are used to lower the stack frequency. Installing a smart skin on a UUV is not an appropriate method, as it must be light and thin. Therefore, we lowered the center frequency using a backing layer at the same time as the PZT stack. The thickness was selected as the thickest among the commercial PZT, and the width was limited to half the wavelength, like the diameter of the cymbal. The electrical input is applied only to the top of the stacked PZT and multiplied by the number of layers in the stack.

### 2.2. Analytical Modeling to Calculate Active Reflection Control Effect Using Multi-Layer Transducer

#### 2.2.1. Algorithm of Active Reflection Control Model

The mass production of smart skin and its attachment to UUV are expensive and time-consuming. Therefore, an analytical active reflection control model was developed to predict the performance of active reflection control [20]. This model consists of an acoustic window, a double-layer receiver sensor, and a stacked piezoelectric transducer. After measuring the external incident signal with the receiver, the signal canceling the reflected wave was transmitted using the phase-delay method. The transmitting and receiving sensors input the characteristics of the previously designed model, and the acoustic window is Rho-c (Aptflex-F21, Precision Acoustics, Dorchester, Dorset, UK). The attenuation loss of Rho-c is 19.8 mdB/cm.

**Figure 6:**
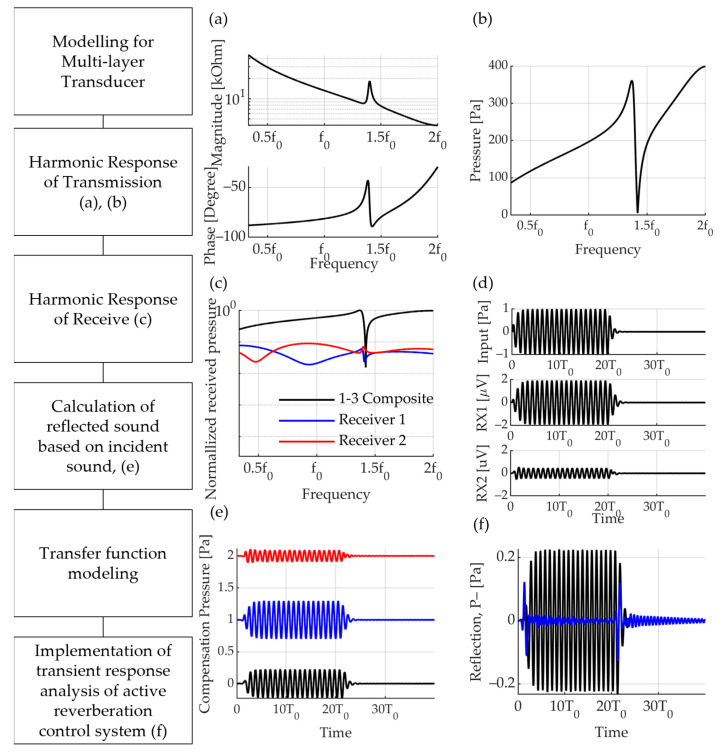
Algorithm of analytical model for active reflection control. (**a**) Electrical impedance; (**b**) harmonic response of transmitter; (**c**) harmonic response of receiver; (**d**) time domain analysis of reception sensitivity according to unit pressure input; (**e**) compensation pressure for reflection control; (**f**) reflection pressure with only passive attenuation (black) and with active reflection control applied (blue).

The algorithm of the active reflection control model consists of six steps (Figure 6). The structure of the smart skin was modelled and we input the material properties of each part. The smart skin has seven layers and is designed with a tungsten block as a backing layer, PZT-5A as a transmitter, two layers of PVDF as a receiver, and three layers of Rho-c as an acoustic window. The front medium of the sensor was water, and the rear medium was air. Frequency response analysis of the reflected wave, reception sensitivity, and transmission sensitivity was performed based on the designed structure and input material properties (Figure 6a,b). Based on the single-input single-output (SISO) model, the process includes transmission and reflection by impedance mismatch and transmission/reception sensitivity using piezoelectric characteristics (Figure 6c). We modelled the transfer function based on a previously designed dynamic system. The transfer function calculates the time response of the dynamic system based on a discrete-time transfer function (Figure 6d). Similarly, it is composed of the SISO system and calculates the reflected wave according to the input signal and the time-domain received signal in RX 1 and RX 2. This model interprets and emits transmitted sound that cancels the reflected wave based on the received signal (Figure 6e). Using the above algorithm, a feedback system was constructed to calculate the performance of the active reflection control model for the input signal (Figure 6f).

#### 2.2.2. BEM Analysis for Omnidirectional Reflection Modeling

Analytical methods for constructing an omnidirectional reflection model include the finite element method (FEM) and the boundary element method (BEM). BEM is widely used in static and dynamic analysis and linear and non-linear analysis as it has the advantage of requiring less data preparation and less computation time. In the case of SONAR, it is a system that measures a very long distance compared to the wavelength, and hence, it consumes a lot of time and money to analyze it using FEM. Therefore, we developed an omnidirectional reflection model that sets the boundary conditions and analyzed them using BEM.

For the omnidirectional reflection model, oblique incidence must be considered in addition to normal incidence. It also interprets reflections from all angles; therefore, the angle between the transmit and receive transducers and each mesh must be calculated. Therefore, it is a function of distance and angle. The distance is calculated from the center point and the positions of the transmitting and receiving transducers. Angle is the angle formed by the normal vector of the transmitting and receiving transducers and the normal vector of each mesh. The reflected signal was calculated based on the Rayleigh–Sommerfeld integral equation:

(1) $$p\left(\vec{r_{p}},t\right)=\frac{1}{2\pi }\int_{s}\;\frac{\cos\theta }{r}\frac{\partial }{\partial t}p\left(\vec{r_{a}},t-\frac{r}{c}\right)dS$$

where $\vec{r_{p}}$ is the position vector of the mesh, r is the distance between the mesh and the elementary area dS ($\vec{r_{a}}$), and c is the speed of sound in the medium. For omnidirectional reflection analysis, the above integral equation was applied for all meshes of the UUV surface.

## 3. Results and Discussion

### 3.1. Characterization of Receiver and Transmitter

#### 3.1.1. Thin Layer Receiver

**Figure 7:**
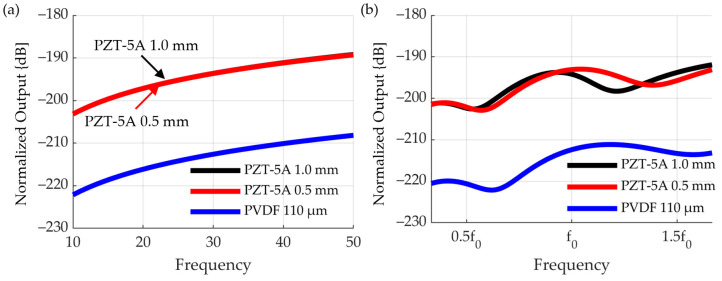
Receiving sensitivity of each material in (**a**) RX 1 (**b**) RX 2.

The main performance indicators of the receiver are the receiver sensitivity, bandwidth, and effect on the transmitted sound. PZT-5A 1.0 mm and 0.5 mm and PVDF 110 μm to be used as receiving sensors all show center frequencies at high frequencies. It is difficult to compare the bandwidths because they operate in the non-resonant mode. Interference with the sensitivity and transmission sound was analyzed using COMSOL, a multiphysics analysis program (Figure 7). The reception sensitivity was taken as a decibel value using the reference pressure (=1 V/μPa).

Within the frequency range, the sensitivity of PZT-5A 1.0 mm and 0.5 mm is, on average, approximately 20 dB higher than that of the PVDF. At low frequencies, RX 1 and RX 2 were similar in sensitivity, but at higher frequencies, RX 1 was more sensitive. This is because the attenuation loss caused by Rho-c was high at high frequencies. There is no significant difference in the reception sensitivity between PZT 1.0 mm and 0.5 mm.

**Figure 8:**
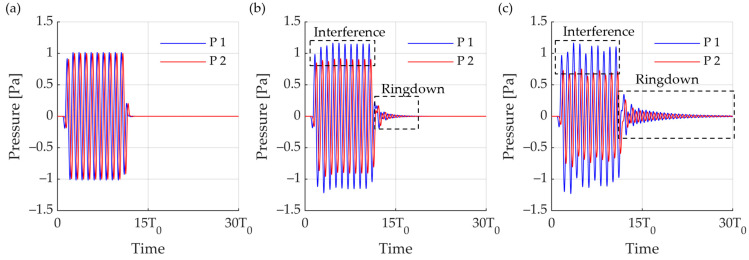
Influence of the material and thickness of the receiving layer on the transmission signal. (**a**) 110 μm PVDF (**b**) 0.5 mm PZT-5A (**c**) 1.0 mm PZT-5A.

To compare the effect on transmission, the waveform was analyzed in the time domain at two points (in the middle between RX 1 and RX 2, and in front of RX 1) (Figure 8). It has been shown that the input signal is also affected and the waveform changes. In addition, a waveform resembling ringdown occurred, and because of frequency analysis through a fast Fourier transform, it resonated in the space between the sensors. Because PZT has a higher acoustic impedance than PVDF, it has been interpreted that reflection of sound waves occurs and affects the transmitted sound. Interference and ringdown were noticeable at 1.0 mm PZT and 0.5 mm PZT. On the other hand, in smart skin using PVDF, the transmit signal maintains the same waveform even after passing through the receiver layer. Therefore, the receiver was selected as PVDF.

#### 3.1.2. Validation of Cymbal Transducer and Stacked PZT Transducer Model

**Figure 9:**
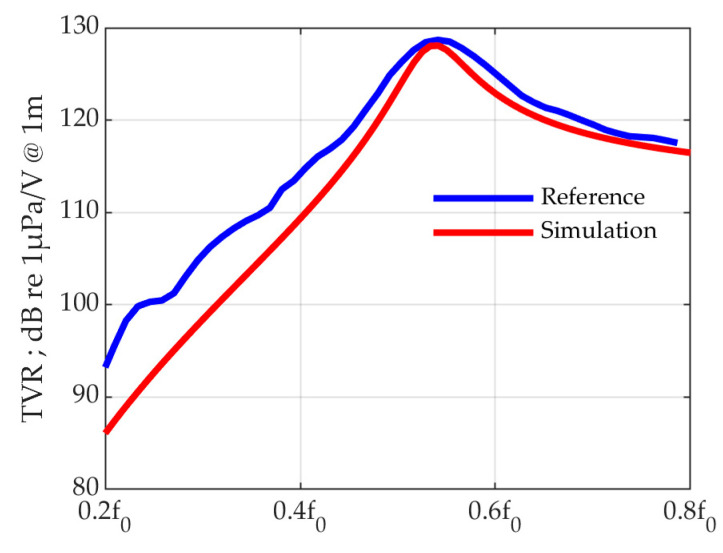
Comparison of the reference model for simulation verification and the finite element analysis model of this paper.

Prior to evaluating the characteristics of the designed transducer, the finite element analysis model of the transducer was compared with existing studies for validation (Table 1). The reference model was designed through an optimization process and manufactured so that the TVR is 0.9 dB smaller, and the bandwidth is similar [21]. Similar characteristics are calculated when comparing the model of this study and the reference model (Figure 9). The center frequency differs by 1.4%, the transmit sensitivity is 0.66 dB, and the FBW is 4.6% lower. In the case of the reference model, the above difference occurs because the coating for waterproofing was considered.

In the case of stacked PZT transducers, it was performed in an existing study [20]. The active reflection control performance was 14 dB in the analysis model and 12 dB in the experiment with the manufactured transducer, which is a 2 dB difference. Through this study, it was verified that the simulation was successfully performed.

#### 3.1.3. Transmitter

**Figure 10:**
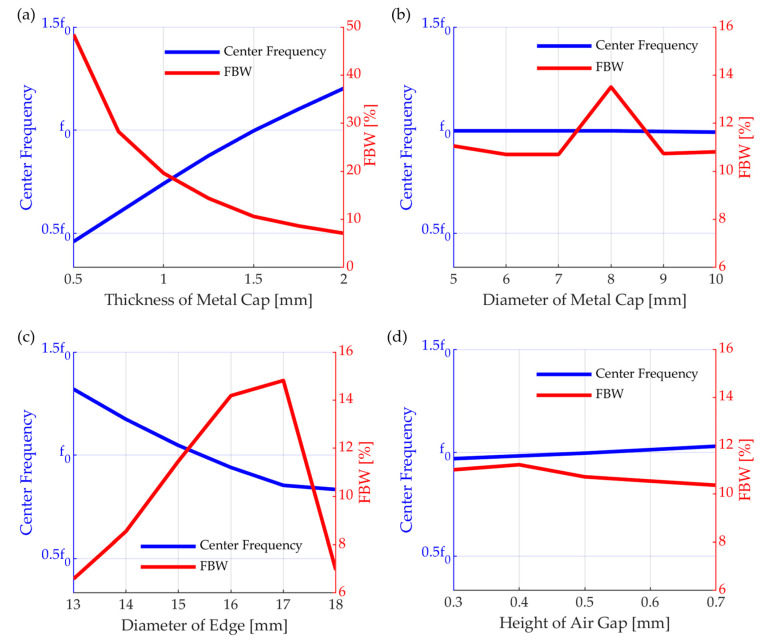
Acoustic properties of a cymbal transducer according to various parameters (**a**) $t_{b}$, (**b**) $r_{a}$, (**c**) $r_{b}$, and (**d**) $h_{A}$.

**Figure 11:**
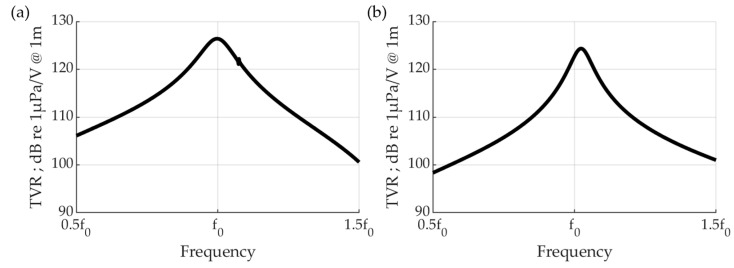
Harmonic response of transmission sensitivity of (**a**) cymbal transducer and (**b**) stacked piezoelectric ultrasound transducer.

The bandwidth of the transmitter affected the transient part of the transmitted wave. Therefore, a wide bandwidth is advantageous for reflectance control. Among the characteristics of the transmitting transducer, the center frequency should be fixed at the target frequency, but the bandwidth should be wide. It is the vibration of the metal that generates sound pressure in the cymbal transducer. Changes in the center frequency and bandwidth according to the parameters were analyzed using the FEM (Figure 10). Because the diameter was limited to half of the wavelength, the center frequency and fractional bandwidth (FBW) were calculated through frequency response analysis while sweeping $t_{b}$, $r_{b}$, $r_{a}$, and $h_{a}$. The greatest influence on the frequency is of the thickness $t_{b}$ of the metal cap and $r_{b}$, i.e., the radius of the metal structure. In the case of $t_{b}$, the center frequency increased proportionally with increasing thickness. However, in terms of bandwidth, a decrease in the thickness is noticeable. The radius of the metal structure is inversely proportional to the center frequency, and the bandwidth is wide for the measured values. In addition, $r_{b}$ determines the radius of the air layer. Therefore, the resonance of the air layer may appear during the analysis. The thickness of the air layer, $h_{a}$, has little effect on the properties, and the top radius of the cap affects the bandwidth. Through the above process, the model with center frequency $f_{0}$ and the widest bandwidth is selected (Table 2, Figure 11). The specific bandwidth is 11.04%, and the center frequency is $f_{0}$. Near $f_{0}$, a change in sensitivity owing to the resonance of the air layer may also appear. The sensitivity at the center frequency is TVR 126.4 dB.

The width of the stacked piezoelectric transducer is limited by the pitch, and the thickness is determined by the frequency. Five PZT plates are stacked. At the center frequency $1.1f_{0}$, the bandwidth is 6.05%, and the TVR sensitivity is 124.3 dB (Table 2, Figure 11). The 6.05% difference in bandwidth does not have a significant effect on the waveform at low frequencies; therefore, a smart skin is configured using a highly sensitive stacked piezoelectric transducer.

### 3.2. Omnidirectional Reflection Model of Active Reflection Control Feedback System

#### 3.2.1. Active Reflection Control Feedback System

**Figure 12:**
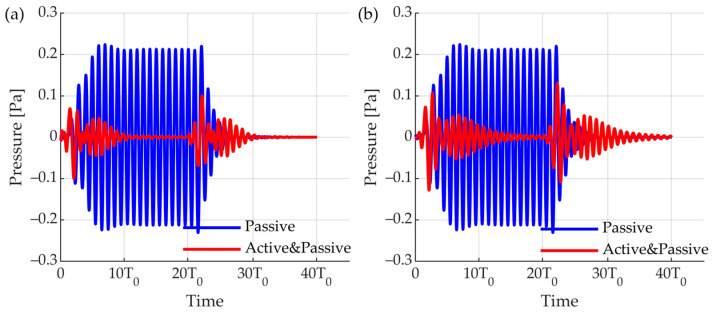
Cancellation effect of active reflection control feedback model. (**a**) Cymbal transducer; (**b**) stack PZT transducer.

Active reflection control was used to design a smart skin consisting of a dual-layer receiver and a single-layer transmitter. For evaluating the characteristics of each sensor, the frequency response calculated through finite element analysis was entered (Figure 11). An active reflection-control feedback system algorithm was constructed to analyze the offset effect of the smart skin. The signal incident from outside was a burst wave with a frequency of $f_{0}$ and 20 cycles and an acoustic wave with a Q factor of 2. The two receiving layers detected a signal and distinguished whether the signal was an incident wave or reflection through a time-delay analysis. If it was analyzed as an incident wave, it sent a signal to the transmitter that cancelled the echo using the feedback algorithm. The sizes of the on and off echoes of the active reflection-control system according to the transmission transducer were compared, and the effect according to the frequency was analyzed (Figure 12). First, we calculated the size of the echo when the feedback system was not used. A signal of 1 Pa was incident, but when considering the attenuation loss owing to Rho-c and the reflectance due to impedance mismatch, a reflection of 0.2 Pa was measured (Figure 12a blue). Because it was a signal with a Q factor of two, transient parts were observed before and after the wave. The passive loss of the system was 13.98 dB at the peak for position 10 $T_{0}$. Because the attenuation loss due to Rho-c was large, the passive system showed an attenuation of more than 10 dB. Reflection attenuation was also determined using an active reflection-control system. The effect of the active reflection control system was analyzed by setting the actuator to a previously designed cymbal transducer and stack PZT. In the case of stack PZT, the signal was canceled by 22.6 dB based on the peak at 10 $T_{0}$, and the attenuation was 35.8 dB based on the incident sound. Even when the RMS pressure was calculated, the reflected sound was attenuated by 7.26 dB, and its effect was 20.46 dB, compared to that of the incident sound. Because the specific bandwidth of the stack PZT was 6.05%, the uncancelled signals remained before and after reflection. In addition, the active reflection-control effect was insignificant, because the phase shift set at $f_{0}$ subsequently did not work with the intended model at frequencies other than $f_{0}$ (Figure 12b). In the case of the cymbal transducer, the sensitivity was lower than that of the stack PZT; however, the specific bandwidth was wide. This feature also appeared in echo control. Compared to the stack PZT, the unoffset signals at the front and back of the echo were shorter. Owing to the lower sensitivity, a higher voltage was required for cancellation. Subsequently, the model was used to calculate the attenuation effect when smart skin was applied to the front of the UUV instead of a single channel.

#### 3.2.2. UUV Modeling for Omnidirectional Reflection Feedback System

**Figure 13:**
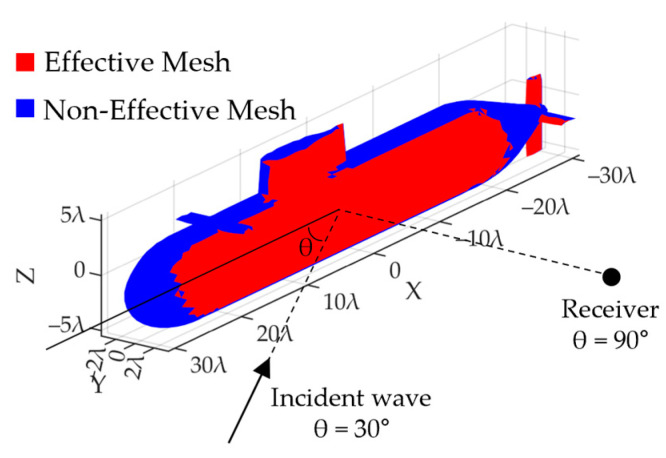
Meshed UUV model diagram. Effective mesh (red) and non-effective mesh (blue) according to angle of incidence and angle of reflection.

An omnidirectional reflection system was modeled to calculate the effect of applying the smart skin to the front of the UUV. The omnidirectional reflection system consists of an external incident wave, a UUV model, and an omnidirectional receiver (Figure 13). When there is an incident wave propagating toward the UUV model from the outside, the receiver measures the sound reflected from each mesh of the UUV. Receiver positioning was performed 360 times at intervals of 1°. The surface of the UUV was meshed with a size of half the wavelength, and the direction vector of each mesh points toward the outside of the UUV. The reflection coefficient of the reflected sound changed according to the angle between the incident wave and the direction vector of each mesh and the angle between the receiver and each mesh. If the incident wave is 45° and the receiver is 135°, the effective mesh of the reflected sound is the same as in the red color in Figure 13.

**Figure 14:**
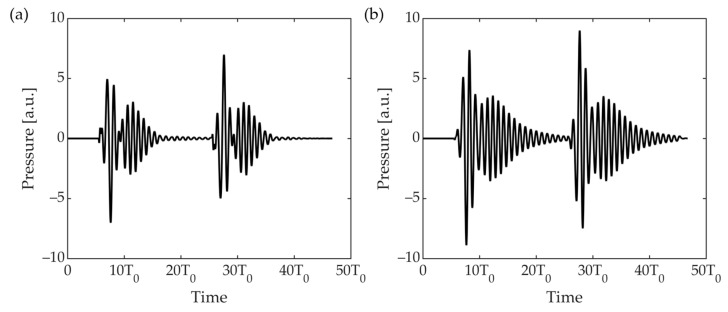
A-Scan that combines raw data received through active reflection control in the active mesh of the UUV model; (**a**) cymbal transducer; (**b**) stack PZT.

The A-scan measured at the receiver can be obtained by calculating the reflection in each mesh using Equation (1) (Figure 14). This is the result obtained by combining the reflections and active reflection control signals of active meshes when the incident angle is 45° and the reception angle is 45°. In other words, it is the sum of the data, such as that observed in Figure 12, according to the angle and distance. Blue lines appeared because of the application of only passive attenuation, excluding the active reflection control system. The black line is the result of applying the reflection coefficient according to the angle, attenuation of Rho-c, and attenuation according to the distance. An active reflection-control system using a cymbal transducer and stack PZT was applied (Figure 13). Based on RMS pressure, attenuations of 27.1 dB (cymbal) and 24.28 dB (stack PZT) occurred, respectively. When the reception angle changed, the active mesh and reflection coefficient also changed. By repeating this process, the peak result at 1–360° was obtained.

The active mesh changed according to the angle of incidence and location of the receiver. As the active mesh is determined according to the direction vector of the mesh, it changes according to the shape of the UUV. The UUV modeled in this study had a hemispherical head and cylindrical hull. The stern was cone-shaped and had a propeller at the end. When viewed from 0°, there was no vertical plane (Figure 14). When the incident angle of the external signal was 180°, the lowest reflection was calculated at a reception angle of 0°. In addition, high echoes were measured at 110° and 150° because the reflection coefficients were high at the front of the submarine and the observation tower, respectively. A peak existed even at 90°, and the signal was reflected from the wings and observation tower of the UUV. Because the UUV had a symmetrical structure, its radiation pattern was also symmetrical.

**Figure 15:**
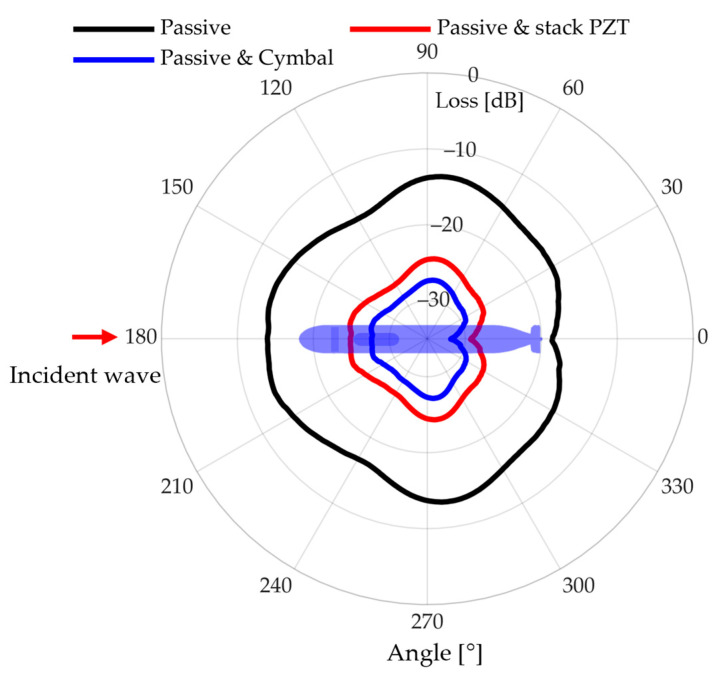
Result of modeling the omnidirectional effect of the active reflection control system- only passive attenuation applied (black), cancellation effect by cymbal transducer (blue), cancellation effect by stack PZT (red).

Red and blue represent the active reflection control effects of the cymbal transducer and stack PZT, respectively (Figure 15). Passive control results in a black shape, and the average absorption is 15.4 dB. The cymbal transducer and stack PZT exhibited additional absorption effects of 28.6 and 26.0 dB, respectively, on average. When a smart skin is applied, we expect to achieve a max attenuation effect of 28.6 dB (Table 3).

## 4. Conclusions

In this paper, an omnidirectional reflection model of UUV applied with an active reflection control system using smart skin was presented. The smart skin is designed with multilayer transducers to enable passive–active reflection control. To reduce the thickness of the smart skin, a cymbal transducer and a stacked PZT transducer were designed, and their sound absorption performance was analytically compared. A feedback model was developed based on the symbol transducer with center frequency $f_{0}$, FBW 11.04% and the stacked PZT transducer with center frequency 1.1$f_{0}$, FBW 6.05%, and the passive-active reflection control effect was calculated. An omnidirectional reflection model was developed to simulate the sound absorption performance when the smart skin is used for the entire UUV. The UUV model was meshed, and the previously developed feedback model was applied to each mesh. When there is an incident sound from the outside, the reflection signal was calculated at 0–360° and executed for each mesh. The sound absorption by passive control is 15.4 dB, the active reflection control by cymbal transducer is 13.2 dB, and the stacked PZT transducer is 10.6 dB. As a result, it can absorb up to 28.6 dB of sound through passive–active reflection control. Omnidirectional reflections of the UUV and attenuation effects were calculated when the smart skin was applied.

## Figures and Tables

**Table 1:** Parameters of reference cymbal transducer.

Cymbal Transducer [21]	
$r_{a}$	2.55 mm
$r_{b}$	7.3 mm
$r_{c}$	10.0 mm
$t_{b}$	0.5 mm
$h_{A}$	0.72 mm
$t_{c}$	1.0 mm
Total thickness	2.5 mm

**Table 2:** Parameters of cymbal transducer and stacked PZT transducer.

Cymbal Transducer		Stacked PZT Transducer	
$r_{a}$	3.5 mm	Width	18 mm
$r_{b}$	10 mm		
$r_{c}$	12.5 mm	$t_{p}$	12.6 mm
$t_{b}$	1.5 mm		
$h_{A}$	0.5 mm	Total thickness	63 mm
$t_{c}$	1.0 mm		
Total thickness	2.5 mm		

$t_{p}:$ thickness of the single PZT.

**Table 3:** Absorption effect of UUV active reflection control simulation using multi-layer transducer.

	Center Frequency	Control Frequency	Passive Control	Passive and Active Control
Cymbal Transducer	$f_{0}$	$f_{0}$	15.4 dB	28.6 dB
Stacked PZT Transducer	$1.1f_{0}$	$f_{0}$	15.4 dB	26.0 dB

## Data Availability

Not applicable.
